# Expression of SARS‐CoV‐2 entry factors, electrolyte, and mineral transporters in different mouse intestinal epithelial cell types

**DOI:** 10.14814/phy2.15061

**Published:** 2021-11-09

**Authors:** Sarah C. Pearce, Panan Suntornsaratoon, Kunihiro Kishida, Arwa Al‐Jawadi, Joshua Guardia, Ian Nadler, Juan Flores, Reilly Shiarella, Madelyn Auvinen, Shiyan Yu, Nan Gao, Ronaldo P. Ferraris

**Affiliations:** ^1^ Department of Pharmacology, Physiology and Neurosciences New Jersey Medical School Rutgers University Newark New Jersey USA; ^2^ Department of Physiology, Faculty of Science Mahidol University Bangkok Thailand; ^3^ Department of Biological Sciences Life Science Center Rutgers University Newark New Jersey USA; ^4^ Present address: USDA‐ARS National Laboratory for Agriculture and the Environment 1015 N. University Blvd. Ames Iowa 50011 USA; ^5^ Present address: Department of Science and Technology on Food Safety Kindai University Wakayama 649‐6493 Japan; ^6^ Present address: Thermo Fisher Scientific 5823 Newton Drive Carlsbad California 92008 USA

**Keywords:** ACE2, calcium, iron, mucosa, TMPRSS, villus

## Abstract

Angiotensin‐converting enzyme 2 (ACE2) and transmembrane proteases (TMPRSS) are multifunctional proteins required for SARS‐CoV‐2 infection or for amino acid (AA) transport, and are abundantly expressed in mammalian small intestine, but the identity of the intestinal cell type(s) and sites of expression are unclear. Here we determined expression of SARS‐CoV‐2 entry factors in different cell types and then compared it to that of representative AA, electrolyte, and mineral transporters. We tested the hypothesis that SARS‐CoV‐2, AA, electrolyte, and mineral transporters are expressed heterogeneously in different intestinal cell types by making mouse enteroids enriched in enterocytes (ENT), goblet (GOB), Paneth (PAN), or stem (ISC) cells. Interestingly, the expression of ACE2 was apical and modestly greater in ENT, the same pattern observed for its associated AA transporters B^0^AT1 and SIT1. TMPRSS2 and TMPRSS4 were more highly expressed in crypt‐residing ISC. Expression of electrolyte transporters was dramatically heterogeneous. DRA, NBCe1, and NHE3 were greatest in ENT, while those of CFTR and NKCC1 that play important roles in secretory diarrhea, were mainly expressed in ISC and PAN that also displayed immunohistochemically abundant basolateral NKCC1. Intestinal iron transporters were generally expressed higher in ENT and GOB, while calcium transporters were expressed mainly in PAN. Heterogeneous expression of its entry factors suggests that the ability of SARS‐CoV‐2 to infect the intestine may vary with cell type. Parallel cell‐type expression patterns of ACE2 with B^0^AT1 and SIT1 provides further evidence of ACE2's multifunctional properties and importance in AA absorption.

## Graphical abstract

SARS‐CoV‐2, the virus responsible for the COVID‐19 pandemic, can infect the small intestine and cause diarrhea, but the specific intestinal cell type(s) expressing its primary receptor ACE2 is unclear. Directed differentiation of mouse enteroids indicated higher expression of ACE2 along the apical membrane of enterocyte‐enriched enteroids compared to that of enteroids enriched in intestinal stem, goblet, or Paneth cells, suggesting greater potential vulnerability of this cell type to infection.

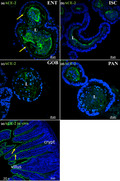

**New and Noteworthy**

SARS‐CoV‐2, the virus responsible for the COVID‐19 pandemic, can infect the small intestine and cause diarrhea, but the specific intestinal cell type(s) expressing its primary receptor ACE2 are unclear. Directed differentiation of mouse enteroids indicated higher expression of ACE2 along the apical membrane of enterocytes compared to that of intestinal stem, goblet, and Paneth cells, suggesting greater potential vulnerability of this cell type to infection.

## 1 INTRODUCTION

Many bacteria and viruses have evolved clever adaptations to breach the gut barrier, including the SARS‐CoV‐2 virus which is causing the ongoing COVID‐19 pandemic. As in other tissues, entry of SARS‐CoV‐2 into intestinal cells depends on the binding of its spike (S) protein to a specific cellular receptor angiotensin‐converting enzyme 2 (ACE2) and on sequential S protein priming by cellular serine proteases. Other than serving as SARS‐CoV‐2 receptor, the main function of ACE2 is to regulate blood pressure, and in the intestine, to interact with absorption of AAs and electrolytes, and with the gut microbiota (Camargo et al., 2020; Koester et al., 2021; Rushworth et al., 2008; Vuille‐dit‐Bille et al., 2015).

Surface expression of ACE2 protein is greatest in mouse and human lung alveolar and small intestinal epithelial cells (Hamming et al., 2004; Zhang et al., 2020). Furin, whose normal function is to activate cellular precursor proteins, initially primes the S protein to mediate viral attachment to mucosal ACE2. The primed S protein is then activated by the host transmembrane serine protease 2 (TMPRSS2) and/or TMPRSS4, leading to viral fusion with the host membrane (Hoffmann et al., 2020; Zang et al., 2020). Although ACE2 was demonstrated to be highly expressed in differentiated absorptive enterocytes, its expression in goblet, Paneth, and other cell types is not known (Lamers et al., 2020; Zhang et al., 2020). Moreover, it is not known which cell type(s) express the other SARS‐CoV‐2 entry factors TMPRSS2, TMPRSS4, furin, and ADAM17 (metallopeptidase domain 17 functioning as tumor necrosis factor α‐converting enzyme) thought to participate in SARS‐CoV‐2 entry or ACE2 secretion (Gheblawi et al., 2020).

Electrolyte and fluid abnormalities are highly correlated with severity of COVID‐19 (Lippi et al., 2020; Wu et al., 2020). Moreover, ACE2 expression is affected by sodium and chloride homeostasis (Post et al., 2020; Rushworth et al., 2008). Intestinal fluid transport depends on ion gradients generated by transporters strategically located in either the apical or basolateral membranes, and in either the crypt or villus regions. The location along the crypt–villus axis of these major electrolyte transporters is well established (Foulke‐Abel et al., 2016; Turner, 2020). Thus, the chloride anion exchanger DRA (dysregulated in adenoma) and the sodium–hydrogen exchanger NHE3 are located mainly in the apical membrane of villus cells, the chloride channel CFTR (cystic fibrosis transmembrane conductance regulator) mainly in the apical membrane of crypt cells, the electrogenic sodium bicarbonate cotransporter 1 (NBCe1) in the basolateral membrane of villus cells, and the sodium‐potassium‐chloride cotransporter 1 (NKCC1) in the basolateral membrane of crypt cells (Jakab et al., 2011) (Foulke‐Abel et al., 2016).

Iron, ferritin, and calcium or their transporters have been linked to SARS‐CoV‐2 and COVID‐19 (Liu et al., 2020; Straus et al., 2020; Vargas‐Vargas & Cortes‐Rojo, 2020; Wagener et al., 2020; Zhao et al., 2019). Specifically, regulation of the body's iron status relies on the appropriate expression of iron transporters/carriers DMT1 (divalent metal transporter 1), IREG1 (iron‐regulated transporter 1 or FPN1), FTL (ferritin light chain), and HEPH (hephaestin), in the small intestine, and on their appropriate distribution between villus and crypt cells (Pietrangelo, 2010). Hypocalcemia is an independent risk factor associated with long‐term hospitalization in patients with COVID‐19 (Wu et al., 2020). Increased nutritional requirements for calcium is met by enhanced expression in the proximal intestine of calcium transporters TRPV6 (transient receptor potential cation channel subfamily V, member 6) and PMCA1 (plasma membrane calcium ATPase 1) (Peng et al., 2018) located in the apical and basolateral membranes, respectively.

Determining the expression of ion transporters in cell types other than enterocytes (ENT) has been difficult because of their low numbers. While absorptive ENTs constitute ~75%–80% and mucus‐secreting goblet (GOB) cells ~5%–10% of all epithelial cells lining the mucosa in vivo, intestinal stem (ISC), enteroendocrine, and Tuft cells each represent <1% of epithelial cells (Cheng & Origin, 1974; Gerbe et al., 2011; Montgomery & Breault, 2008; Umar, 2010). Secretory Paneth cells (PAN, ~5% of cells) migrate down to the crypt region where stem cells are also located. Because ENTs predominate, many functions of the small intestine are ascribed primarily to this cell type, and there has been no study using enteroids enriched in specific cell types to determine the expression of SARS‐CoV‐2 entry factors as well as major electrolyte and mineral transporters in non‐enterocytes. We used mouse enteroids as no standardized protocols exist to reliably direct the differentiation of the major epithelial cell types of human intestine, whereas directed differentiation of murine ISC have been well established (Kishida et al., 2017; Pearce et al., 2018). ACE2 and TMPRSS2 proteins are highly conserved among vertebrates suggesting that determinants of cell type expression may be similar among humans, mice, and other mammals (Damas et al., 2020; Lam et al., 2020; Li et al., 2020).

In this study, we tested the hypothesis that SARS‐CoV‐2 entry factors, electrolyte transporters, ACE2‐associated AA transporters, calcium as well as iron transporters are distributed heterogeneously among the various intestinal cell types. We directed the differentiation of small intestinal crypt precursors to enteroids enriched in ENTs (~90% of all cells per enteroid), GOB cells (~80%), PAN (~65%), or stem cells (~90%) (Kishida et al., 2017; Pearce et al., 2018), then used these enteroids to probe for expression of these transporters and SARS‐CoV‐2 entry factors.

## 2 METHODS

### 2.1 Animals

All procedures conducted in this study were approved by the Institutional Animal Care and Use Committee, New Jersey Medical School, Rutgers University. Crypts from the proximal intestine were isolated from 6‐ to 8‐week‐old wild‐type male mice (WT; Taconic Laboratories, Hudson, NY).

### 2.2 Directed differentiation of intestinal enteroids

In vitro differentiation of ISCs in isolated crypt fragments can be controlled by taking advantage of binary cell fate decisions regulated by the Wnt‐ and Notch‐signaling pathways (Pearce et al., 2018; Yin et al., 2014). Briefly, we used various combinations depending on the desired enteroid outcome, the following small molecules: 3 μM CHIR99021 (Wnt activator; Stemgent, Cambridge, MA), 2 mM valproic acid (Notch activator; Tocris Bioscience, United Kingdom), 2 μM C59 (Wnt inhibitor; Stemgent, Cambridge, MA), and 10 μM DAPT (Notch inhibitor; Tocris Bioscience, United Kingdom) that were added to the basic culture medium containing epidermal growth factor (50 ng/ml; Life Technologies), murine R‐spondin‐1 (a Wnt modulator, 500 ng/ml; PeproTech, Rocky Hill, NJ), and murine Noggin (ligands of TGF‐β family, 100 ng/ml; PeproTech). The type of enteroid produced is confirmed by determining the expression of biomarker genes known to be expressed specifically in ISC, PAN, ENT, or GOB cells. If crypt precursors are grown only in the basic culture media, the enteroids produced would consist of all cell types in proportions similar in vivo, and are referred to as typical (TYP) enteroids. ENT, GOB, and PAN enteroids were differentiated from ISC enteroids and characterized at ~3 days after initiation of differentiation when biomarker expression becomes stable (Kishida et al., 2017).

Comparative mRNA and protein expression levels of characteristic biomarkers of ISC, ENT, GOB, and PAN cells confirmed that directed enteroids were highly enriched in specific cell types and were similar to our previous work (Kishida et al., 2017; Pearce et al., 2018).

### 2.3 Dedifferentiation

Crypts were isolated and differentiated into ENT enteroids as described above. After 72 h in ENT‐enriching media, ENT enteroids were dedifferentiated (dENT) by adding ISC‐enriching media containing 6 μM CHIR99021 + 2 mM VPA for 36 h (Pearce et al., 2018).

### 2.4 Real‐time PCR

Total RNA was extracted from intestinal enteroids using a commercially available kit (RNeasy Micro, Qiagen). Real‐time PCR using Mx3000P (Stratagene, La Jolla, CA) was used to analyze cDNA using Maxima SYBR Green (Thermo Fisher Scientific, Grand Island, NY). Primer sequences (Integrated DNA Technologies [IDT; Coralville, IA]) are listed (Table 1). All samples were standardized to β‐actin expression.

**TABLE 1:** Primer sequences used in PCR

Gene name	Sense (5’‐3’)—Forward	Antisense (5’‐3’)—Reverse
ACE2 (Angiotensin‐converting enzyme 2)	TCCATTGGTCTTCTGCCATCC	AACGATCTCCCGCTTCATCTC
ACTIN (β—actin) (*Actb*)	TTGTTACCAACTGGGACGACATGG	CTCGGGTGTTGAAGGTCTCAAACA
ADAM17 (Disintegrin & metallopeptidase domain 17)	AGCTGCAGCGTCAGAGC	CAGCACTGTCACCAGGAAC
B^0^AT1 (Sodium‐dependent neutral amino acid transporter 1) (*Slc6a19*)	TCACCTGTGTGGGCTTTTGT	CCTCGAACACCAGAAGGATG
CFTR (Cystic fibrosis transmembrane conductance regulator)	AAGGCGGCCTATATGAGGTT	AGGACGATTCCGTTGATGAC
DMT1 (Divalent metal transporter) (*Slc11a2*)	GAGCAGTGGCTGGATTTAAG	CGGTGACATACTTCAGCAAG
DRA (Downregulated in Adenoma) (*Slc6a3*)	TTCCCCTCAACATCACCATCC	GTAAAATCGTTCTGAGGCCCC
FTL (Ferritin‐light‐chain)	CTTCCAGGATGTGCAGAAG	ATCCAAGAGGGCCTGATT
Furin	CAGCGAGACCTGAATGTGAA	CAGGGTCATAATTGCCTGCT
HEPH (Hephaestin)	GCAGTGGAACTATGCTCC CAA	CAGCCTGTAACAGTGGTC CTA
IREG1 (Ferroportin‐1)	GGGTGGATAAGAATGCCAGAC	CCTTTGGATTGTGATCGCAGT
LYZ (Lysozyme)	ATGGCTACCGTGGTGTCAAG	CGGTCTCCACGGTTGTAGTT
MUC2 (Mucin‐2)	CTTCTGTGCCACCCTCGT	TTCGGGATCTGGCTTCTT
NHE3 (Na‐H Antiporter 3) (*Slc9a3*)	TGCCTTGGTGGTACTTCT GG	TCGCTCCTCTTCACCTTCAG
NKCC1 (Na‐K‐Cl symporter)	CAAGGGTTTCTTTGGCTAT	TCACCTGAGATATTTGCTCC
NBCe1 (Sodium bicarbonate cotransporter)	AGCCATCTTCTGCCTTTTTG	TTCTTCTGTGAAACGGGTGA
OLFM4 (Olfactomedin 4)	GCCACTTTCCAATTTCAC	GAGCCTCTTCTCATACAC
PMCA1 (Plasma membrane Ca^2+^ ATPase 1)	TGACGATGAACAGGATGACG	CCAAGAGAAACCCCAACAAG
SI (Sucrase‐isomaltase)	ATCCAGGTTCGAAGGAGAAGCACT	TTCGCTTGAATGCTGTGTGTTCCG
SIT1 (Sodium‐dependent imino acid transporter 1) (*Slc6a20*)	TCATTGTGCTGGTAGAGACCAT	CCCGAAGGTCACTTTCAAAT
TMPRSS2 (Mucosa‐specific serine protease 2)	GAGAACCGTTGTGTTCGTCTC	GCTCTGGTCTGGTATCCCTTG
TMPRSS4 (Mucosa‐specific serine protease 4)	CTGCCTTGACTGTGGAAAG	GCTGCTTGTTGTACTGGATG
TRPV6 (Transient receptor potential cation channel subfamily V member 6, Intestinal)	GCTGATGGCTGTGGTAATTCT	GGGATCCTCTGTCTGGAAAA

### 2.5 Staining

Enteroids were fixed in 4% paraformaldehyde, gently centrifuged (200 g), and processed following earlier work (Pearce et al., 2018). Samples were subsequently de‐paraffinized and subjected to antigen retrieval by 10 min incubation in 10 mM boiling sodium citrate buffer (pH 6.0). Immunostaining was then performed using antibodies and dilution factors (Table 2) as previously described (Kishida et al., 2017).

**TABLE 2:** Dilution factors and source of antibody

Protein	Company	Cat #	Dilution
Angiotensin‐converting enzyme 2 (ACE‐2)	Abcam	Ab108252	1:50
Chromogranin A (CHGA)	Santa Cruz Biotechnology	sc‐13090	1:200
Lysozyme (LYZ)	BioGenex	AR0245R	None
Mucin‐2 (MUC2)	Santa Cruz Biotechnology	sc‐15334	1:200
Na‐K‐Cl symporter (NKCC1)	Santa Cruz Biotechnology	sc‐21547	1:100
Olfactomedin‐4 (OLFM4)	Cell Signaling	D6Y5A	1:1000
Sucrase‐isomaltase (SI)	Santa Cruz Biotechnology	sc‐27603	1:500
Transmembrane serine protease 2 (TMPRSS2)	Santa Cruz Biotechnology	sc‐515727	1:50

### 2.6 Statistical analyses

Data are presented as means ± SEM. To analyze the significance of enteroid type, genotype, or enteroid age, either a one‐way or multi‐way ANOVA was used with a Tukey’s test for multiple comparisons. Differences were considered significant at *p* ≤ 0.05 (GraphPad Prism, GraphPad Software, San Diego, CA).

## 3 RESULTS

### 3.1 Expression of SARS‐CoV‐2 entry cofactors in small intestinal enteroids

**FIGURE 1:**
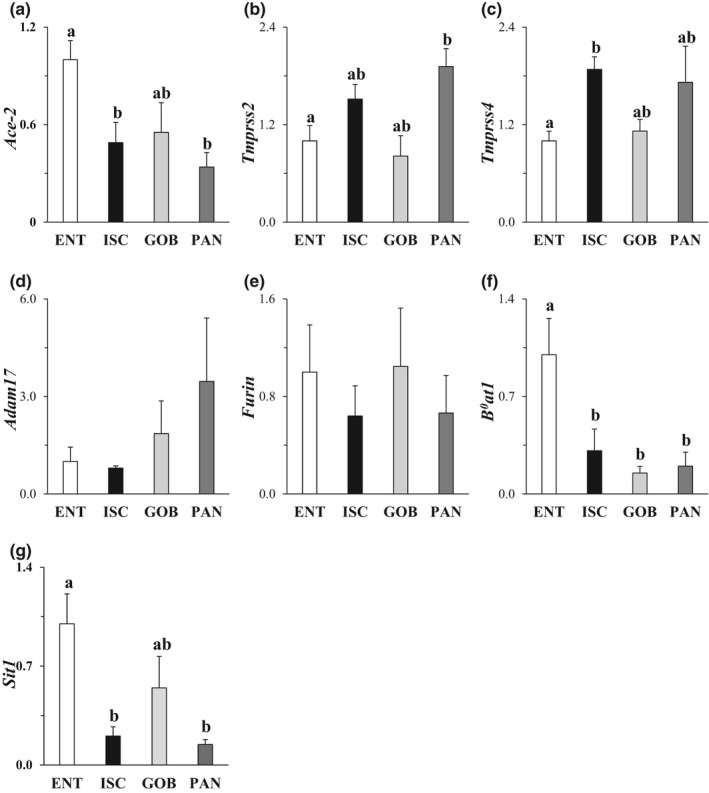
Effect of cell type on mRNA expression of SAR‐CoV‐2 entry factors. (a) ACE2 (angiotensin‐converting enzyme 2), (b,c) TMPRSS2 and 4 (mucosa‐specific serine proteases 2 and 4), (d) ADAM17 (a disintegrin and metallopeptidase domain 17), (e) Furin proteinase, (f) B^0^AT1 (neutral amino acid transporter) and (g) SIT1 (sodium‐dependent imino acid transporter). Levels of mRNA in all enteroids were normalized to those in ENT enteroids (=1.0). Analysis by one‐way ANOVA, *p* ^a,b^ ≤ 0.05, *n* = 5–8

ACE2 expression was 2‐ to 2.5‐fold greater in ENT compared to other enteroids (Figure 1a). There was modest but still significant expression in ISC, GOB, and PAN enteroids. This result is consistent with single‐cell analysis of ileum from 13 children that revealed ACE2 in absorptive and “crypt‐based” (perhaps transitory) enterocytes but reduced and still significant expression in all other cell types (Zhang et al., 2020). The expression of TMPRSS2 and TMPRSS4 was modestly higher in PAN and ISC, (Figure 1b and c). Levels of furin mRNA were similar among the four types of intestinal enteroids, in accordance with their known ubiquitous expression in all cells (Figure 1d). The expression of ADAM17 known to shed ACE2 from cell membranes was also similar among cell types (Figure 1e). The neutral AA transporter B^0^AT1 was threefold higher in ENT compared to ISC enteroids, and fivefold higher than PAN enteroids (Figure 1f). Likewise, the imino acid transporter SIT1 expression (Figure 1g) was greatest in ENT by 2‐ to 4‐fold compared to that in other cells types. Thus, ACE2 expression mirrors that of B^0^AT1 and SIT1.

**FIGURE 2:**
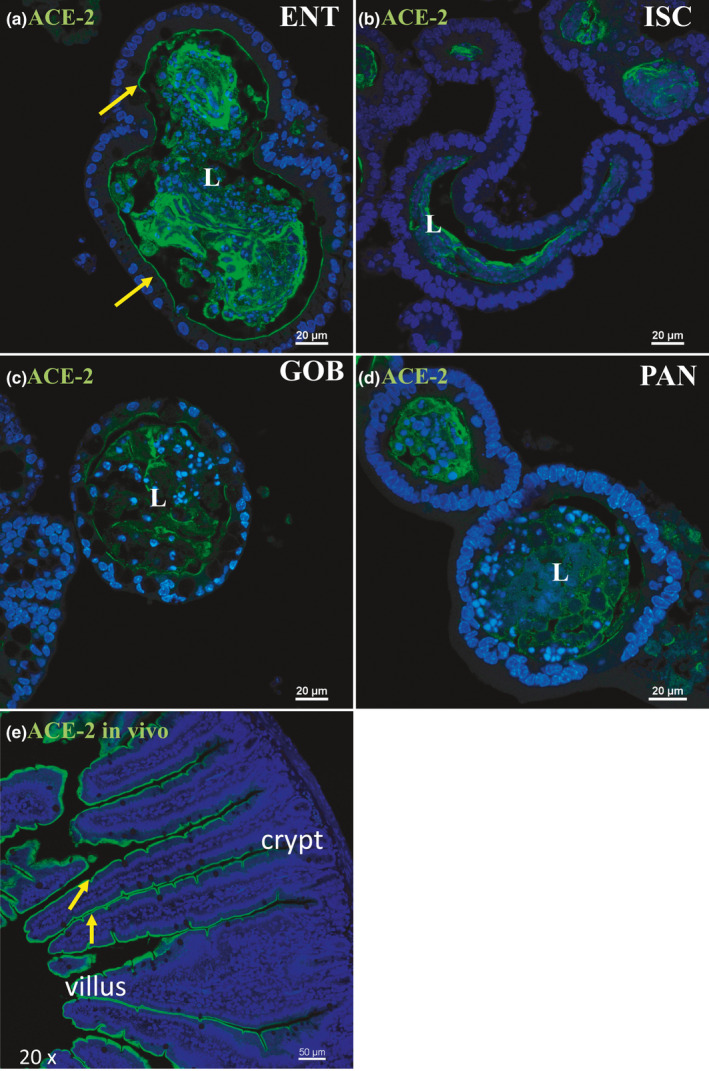
Immunofluorescence staining of ACE2 in enteroids enriched in different cell types, and in vivo. Nuclei are stained blue. Enteroids were probed with ACE2 antibody (green) in ENT (a), ISC (b), GOB (c), and PAN (d) enteroids. ACE2 is present along the apical membrane of ENT but not in ISC, GOB, and PAN enteroids. ACE2 accumulation in the lumen (L), especially in ENT enteroids, suggests that this protein is likely secreted or shed from the cells as previously demonstrated in other organ systems (Gheblawi et al., 2020). Representative enteroids are shown at 60X magnification. Scale bars, 20 μm. In vivo, ACE2 is found lining the villus, but not crypt, region (e). Image is shown at 20X magnification, scale bar, 50 μm. Images are representative of several enteroids or sections from two mice

**FIGURE 3:**
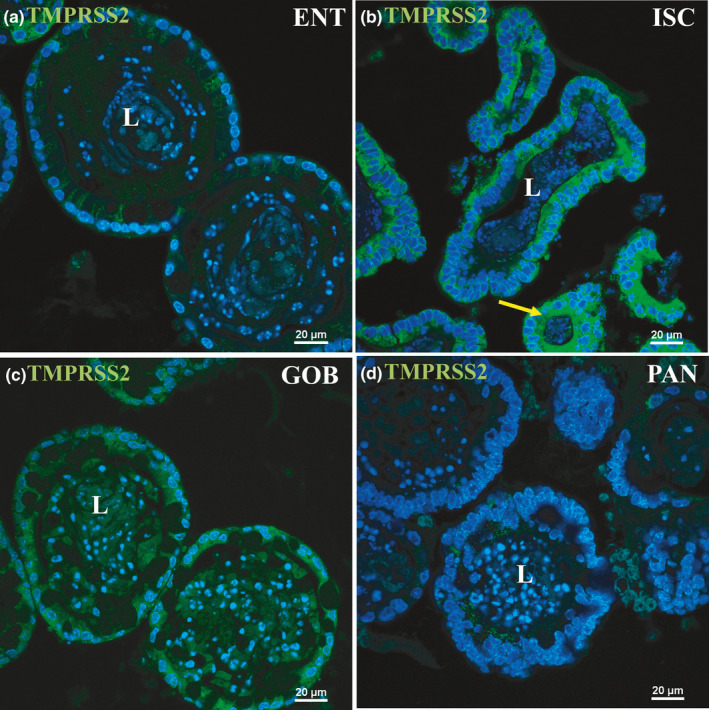
Immunofluorescence staining of TMPRSS2 in enteroids enriched in different cell types. Nuclei are stained blue. Enteroids were probed with TMPRSS2 antibody (green) in ENT (a), ISC (b), GOB (c), and PAN (d) enteroids. TMPRSS2 is present (arrow) along the apical membrane and in the cytosol of ISC and, more modestly, of GOB enteroids. Representative enteroids are shown at 60X magnification. Scale bars, 20 μm. Images are representative of several enteroids from two mice

Protein expression of ACE2 via immunofluorescence‐matched mRNA expression closely with highest expression in ENT enteroids (Figure 2a, arrows). Minimal ACE2 protein appeared in the apical membranes of ISC, GOB, or PAN enteroids (Figure 2b–d). Luminal accumulation of ACE2 in enteroids, especially in ENT, indicates that this enzyme is also secreted or shed by enterocytes as was found for ACE2 in other organ systems (Gheblawi et al., 2020). This distribution matches exactly the immunocytochemistry of ACE2 distribution along the crypt–villus axis of mouse intestinal tissue (Figure 2e). TMPRSS2 protein expression via immunofluorescence was modest in ENT, GOB, and PAN and highest in ISC enteroids (Figure 3a–d, arrow). Differences between mRNA and protein expression of TMPRSS2 in some cell types suggest posttranscriptional regulation as previously shown (Deng et al., 2011). Negative controls without primary antibodies were devoid of significant immunofluorescence (not shown).

### 3.2 Electrolyte transporters

**FIGURE 4:**
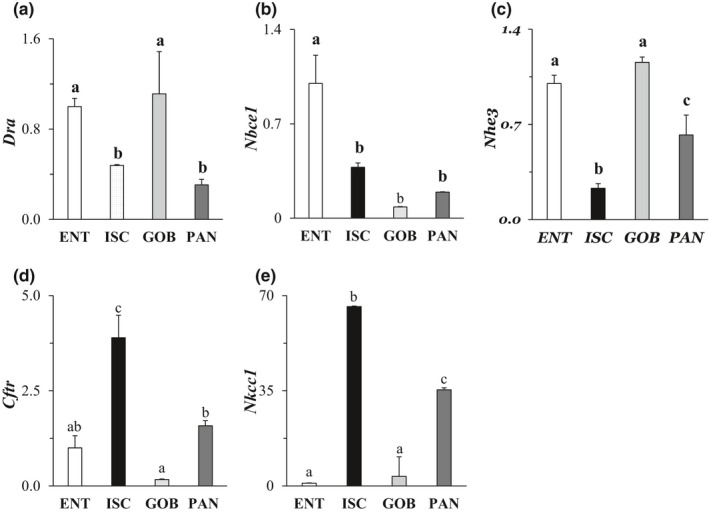
Effect of cell type on mRNA expression of representative intestinal ion transporters. (a) DRA (downregulated in Adenoma, a chloride‐anion exchanger), (b) NBCe1 (sodium‐bicarbonate co‐transporter 1), (c) NHE3 (sodium hydrogen exchanger 3), (d) CFTR (cystic fibrosis transmembrane conductance regulator, a chloride channel), and (e) NKCC1 (sodium‐potassium‐chloride cotransporter). Levels of mRNA in all enteroids were normalized to those in ENT enteroids (=1.0). Analysis by one‐way ANOVA, *p* ^a,b^ ≤ 0.05, *n* = 4–6

Classical transport models depict ion transporters in villus cells to drive net fluid absorption from lumen to blood (Thiagarajah et al., 2018). The mRNA levels of a relevant transporter, DRA (absorbs chloride from the lumen in exchange for bicarbonate; located in the apical membrane of villus cells (Foulke‐Abel et al., 2016)), were ~twofold higher in ENT and GOB enteroids compared to those in ISC and PAN (Figure 4a). The DRA expression also seemed higher in differentiated compared to undifferentiated enteroids (Foulke‐Abel et al., 2016). Similarly, NBCe1 (absorbs bicarbonate from the blood in exchange for chloride; in the basolateral membrane of villus cells (Jakab et al., 2011) was most highly expressed in ENT enteroids, with mRNA levels ~2.5‐ to 10‐fold greater than those of other enteroids (Figure 4b). The expression of another transporter known to contribute to fluid transport, NHE3 (located mainly in the apical membrane of villus cells (Jakab et al., 2011)) seemed to be present mainly in differentiated cell types. NHE3 (absorbs sodium from the lumen in exchange for hydrogen) was significantly lower in ISC enteroids (more than twofold) compared to ENT, GOB, and PAN enteroids (*p* < 0.05; Figure 4c).

**FIGURE 5:**
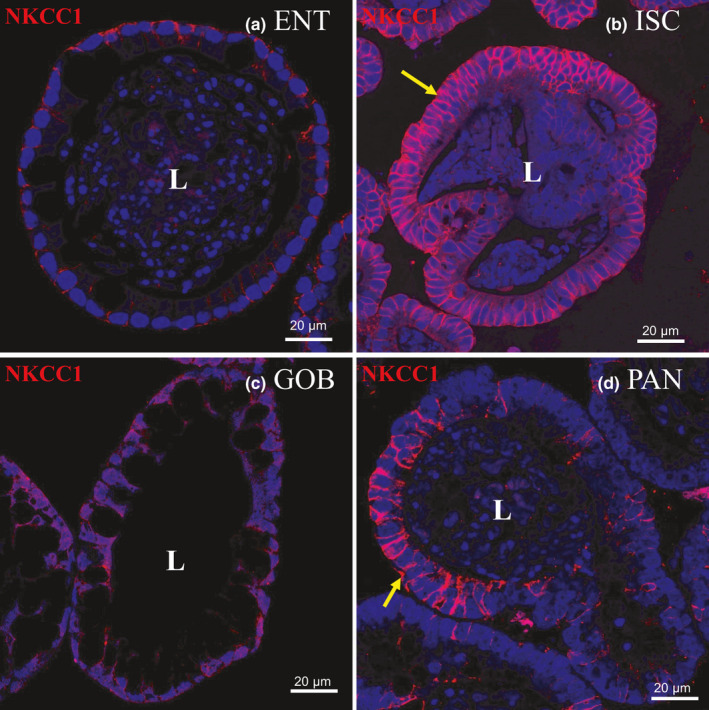
Immunofluorescence staining of NKCC1 in enteroids enriched in different cell types. Nuclei are stained blue. Enteroids were probed with NKCC1 antibody (red) in (a) ENT, (b) ISC, (c) GOB, and (d) PAN enteroids. Representative enteroids are shown at 60X magnification. NKCC1 is present (arrows) along the basolateral membrane of ISC and of PAN enteroids. Scale bars, 20 μm. Images are representative of several enteroids from two mice

In contrast, ion transporters in crypt cells are thought to drive fluid secretion from blood to lumen (Thiagarajah et al., 2018). CFTR (secretes chloride through the apical membrane of crypt cells (Foulke‐Abel et al., 2016; Jakab et al., 2011) was found mostly (by 1.5‐ to 4‐fold) in crypt‐dwelling cell types, ISCs and PAN cells (Figure 4d)). In humans, however, CFTR protein expression seemed similar in differentiated and undifferentiated enteroids (Foulke‐Abel et al., 2016). The mRNA levels of NKCC1 (sodium and potassium cotransport into the cell from the blood with chloride, in the basolateral membrane of crypt cells (Jakab et al., 2011) were greatest in ISC followed closely by PAN enteroids. NKCC1 was >20‐fold higher in ISC than in ENT and GOB enteroids (Figure 4e)). Distribution of NKCC1 expression by immunocytochemistry parallels that of mRNA and was greatest in the ISC and PAN enteroids (Figure 4e). The mRNA distribution among cell types was confirmed with NKCC1 protein that clearly occupied the basolateral membrane of ISC and PAN cell types that line the crypt. There was little immunocytochemical expression of NKCC1 in GOB enteroids where this transporter is also localized basolaterally, and virtually no expression in ENT enteroids (Figure 5).

### 3.3 Iron and calcium transporters

**FIGURE 6:**
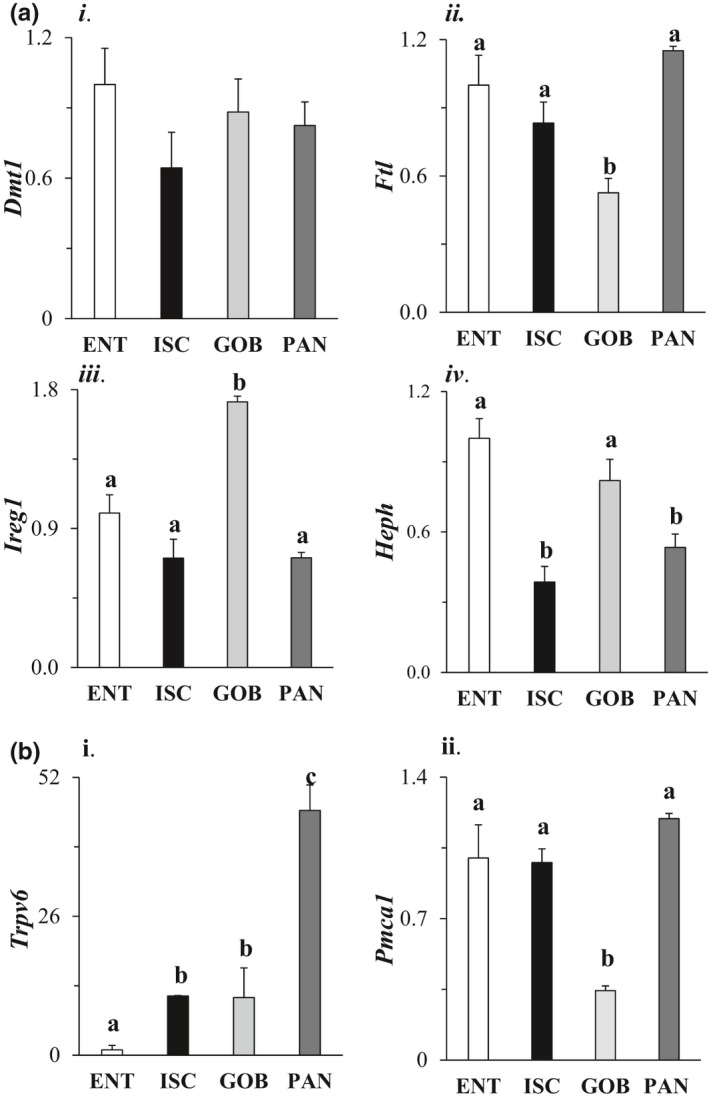
Effect of cell type on mRNA expression of members of the intestinal iron (a) and calcium (b) transport systems. Genes analyzed include (i) DMT1 (divalent metal transporter 1), (ii) FTL (ferritin light chain), (iii) IREG1 (iron regulatory protein 1), and (iv) HEPH (hephaestin). Levels of mRNA in all enteroids were normalized to that in ENT enteroids (=1.0). Analysis by one‐way ANOVA, *p* ^a,b^ ≤ 0.05, *n* = 4–6. For calcium ion transporters, the following were analyzed: (i) TRPV6 (transient receptor potential cation channel vallinoid family 6), and (ii) PMCA1 (plasma membrane calcium ATPase 1). Levels of mRNA in all enteroids were normalized to that in ENT enteroids (=1.0). Analysis by one‐way ANOVA, *p* ^a,b,C^ ≤ 0.05, *n* = 4–6

By one‐way ANOVA, iron and calcium transporters were heterogeneously distributed among different intestinal cell types (*p* < 0.05 in all cases), with the exception of DMT1 which transports iron from the lumen into the cytosol. DMT1 was expressed homogeneously among four cell types (Figure 6a, i). Expression of FTL which regulates cytosolic iron levels was significantly (also twofold) lower in GOB enteroids compared to that in other enteroid types (Figure 6a, ii). Expression of IREG1 which exports iron from the cytosol to the blood was greatest in GOB enteroids (Figure 6a, iii). mRNA expression of hephaestin (HEPH) which contributes to basolateral export of iron to the blood was highest in ENT enteroids (Figure 6a, iv). In general, intestinal mucosal factors that mediate iron absorption were usually highly expressed in ENT enteroids.

Interestingly, PAN enteroids had significantly much higher mRNA expression of TRPV6 compared to that in other cell types (Figure 6b, i). The mRNA levels of the basolateral PMCA1 which actively exports calcium from the cytosol to the blood were lowest in GOB enteroids (2.5‐fold; Figure 6b, ii).

### 3.4 Dedifferentiation and expression of electrolyte transporters

**FIGURE 7:**
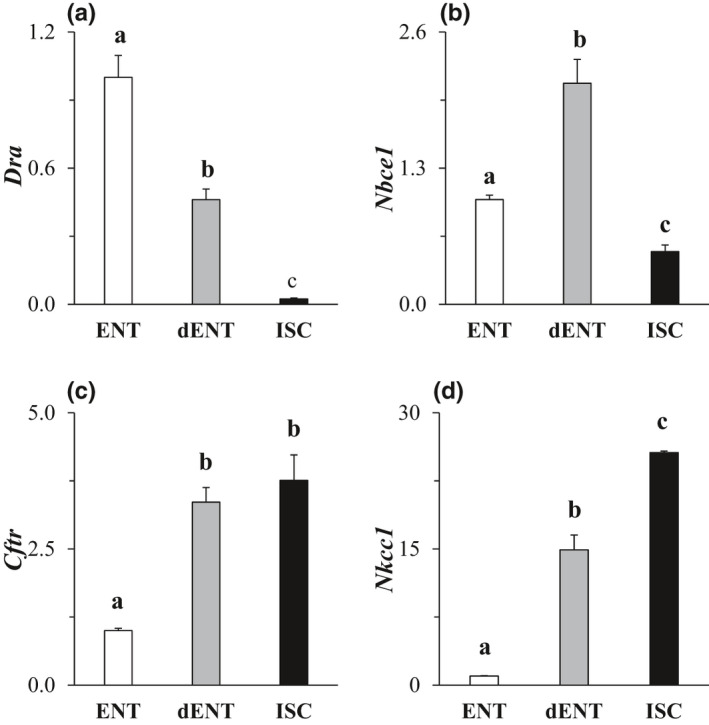
Expression of ion transport mRNA in dedifferentiated enterocyte enteroids. Genes analyzed were (a) DRA, (b) NBCe1, (c) CFTR, and (d) NKCC1. Within each gene, all treatments are normalized to ENT (=1.0) for both dENT and ISC enteroids. Data were analyzed with a one‐way ANOVA. *p* ^a,b,c^ ≤ 0.05, *n* = 3

Because of remarkable differences between ISC and differentiated cell types, we determined whether ISC characteristics were reacquired if mature cell types were forced to dedifferentiate. Once ENT enteroids reached full differentiation as tracked by biomarker expression (Pearce et al., 2018), we were able, by reintroducing growth factors that promoted stemness and proliferation, to initiate and maintain dedifferentiation, and thus could evaluate the properties of dedifferentiated ENT (dENT) enteroids. Of specific interest was NKCC1 expression which differed by ~50‐fold between crypt‐ and villus‐dwelling cell types (Figure 7). In general, when ENT enteroids were forcibly dedifferentiated, the ENT phenotype tended to revert to the ISC phenotype (compare Figure 4 and Figure 7). It is interesting to note that expression of DRA, NKCC1, and CFTR in dENT enteroids was intermediate between those of ISC and ENT. However, there was a transient increase in expression of NBCe1 during the process of de‐differentiation.

## 4 DISCUSSION

Our main finding is that the distribution pattern of mRNA expression of SARS‐CoV‐2 entry cofactors among different intestinal cell types was modestly heterogeneous, with ACE2 being significantly higher in ENT, and with TMPRSS2 as well as TMPRSS4 being greater in ISC. The expression of ACE2 was matched exactly by that of its co‐regulated AA transporters, providing further evidence of the nutritional importance of ACE2. In contrast, expression of major intestinal electrolyte transporters showed marked differences among cell types depending primarily on their location along the villus crypt axis (Table 3). Forced dedifferentiation of ENT enteroids showed enterocytes reacquiring stem cell characteristics of electrolyte transporters.

**TABLE 3:** mRNA expression of SARS‐CoV‐2 entry factors as well as amino acid, ion, and mineral transporters in different intestinal cell types

	ENT	ISC	GOB	PAN
SARS‐CoV‐2 entry				
ACE2	++++	++	+++	++
TMPRSS2	+++	+++	+++	++++
TMPRSS4	++	+++	++	+++
FURIN	+++	+++	+++	+++
ADAM17	+++	+++	+++	+++
Amino acid transporters				
B^0^AT1	++++	++	++	++
SIT1	++++	++	+++	++
Ion transporters				
DRA	++++	++	++++	++
NBCe1	++++	++	+	+
NHE3	++++	+	++++	++
CFTR	++	++++	+	+++
NKCC1	+	++++	+	+++
Mineral transporters				
DMT1	+++	+++	+++	+++
FTL	++++	++++	++	++++
IREG	++	++	++++	++
HEPH	++++	++	+++	++
TRPV6	+	++	++	++++
PMCA1	++++	++++	++	++++

Expression relative to that in organoids enriched in enterocytes (ENT).

ISC, intestinal stem cells; GOB, goblet cells; PAN, Paneth cells; +, no or low expression; ++, modest; +++, high; ++++, very high.

### 4.1 Expression of SARS‐CoV‐2 entry factors in different intestinal cell types

The prevalence of GI symptoms and the numerous reports of SARS‐CoV‐2 RNA in fecal specimens as well as the presence of the viral nucleocapsid protein in the cytoplasm of duodenal cells of COVID‐19 patients (Xiao et al., 2020) suggest that the small intestine can be a major route of viral entry into the human body. SARS‐CoV‐2 cannot enter BHK‐21 cells unless these are transfected with ACE2, and antiserum raised against ACE2 blocked SARS‐CoV‐2 entry (Hoffmann et al., 2020), suggesting that ACE2 is required for viral binding to the mucosal surface. Since ACE2 and TMPRSS2 in mice and humans are already highly expressed throughout the small intestine and the colon (Burgueno et al., 2020; Camargo et al., 2020), modest differences in the expression of ACE2 among different intestinal cell types suggest that the S protein of SARS‐CoV‐2 has the potential to bind to all intestinal cells.

Cross‐species sequence comparisons reveal that only the sequence of the spike protein involved in virus binding is highly variable, resulting in significant differences in SARS‐CoV‐2 vulnerability among mammals (Damas et al., 2020; Lam et al., 2020; Li et al., 2020). Otherwise, the sequence of ACE2 which has other important physiological functions, is highly conserved among vertebrates. This suggests that, while the S protein‐dependent affinity for SARS‐CoV‐2 may differ among mammals, ACE2 regulation of AA and electrolyte transporters, its interactions with gut microbiota, and its expression among intestinal cell types are likely similar (Jia et al., 2020). Thus, there is now an increasing number of studies using mouse genetics involving ACE2 deletion and overexpression, for further understanding of ACE2 biology and for the investigation of ACE2 not only in the pathogenesis and treatment of COVID‐19 but also in human AA nutrition (Jia et al., 2020; Johansen et al., 2020).

The ubiquitous nature of furin is confirmed by its relatively homogeneous expression among small intestinal cell types. TMPRSS2 as well as the other proposed host receptors/facilitators of intestinal SARS‐CoV‐2 entry, ADAM17 and TMPRSS4 (Zang et al., 2020) also are highly expressed with modest differences among cell types. The significant expression of these proteases suggests that the S protein can be cleaved and primed to facilitate fusion between intestinal and SARS‐CoV‐2 membranes in all cell types.

### 4.2 ACE2 and intestinal amino acid transport

Expression levels of ACE2, B^0^AT1, and SIT1 are all greatest in ENT enteroids. This similarity in patterns of expression among cell types of functionally unrelated brush border proteins is due to B^0^AT1 and SIT1 regulation by ACE2 which heterodimerizes with either transporter (Camargo et al., 2020). This association with ACE2 by SIT1 or B^0^AT1 is required for AA absorption. Expression of B^0^AT1 in rodent intestine depends on the presence of ACE2, acting as a chaperone (Vuille‐dit‐Bille et al., 2015), and both ACE2 and B^0^AT1 expression levels increase with levels of dietary protein (Jando et al., 2017). ACE2 has been tightly linked to Hartnup's disease, and regulates neutral AA transport in both rodents and humans (Jando et al., 2017; Kowalczuk et al., 2008). Although these ACE2:SIT1 or ACE2: B^0^AT1 heterodimers have been proposed to function as binding sites for the SARS‐CoV‐2 spike proteins (Guney & Akar, 2021), there is really no clear evidence that this association affects the role of ACE2 in viral infection. However, possible loss of B^0^AT1‐mediated AA absorption due to SARS‐CoV‐2 may contribute to gastrointestinal symptoms observed during COVID‐19, including diarrhea (Barbosa da Luz et al., 2020).

### 4.3 Distribution of electrolyte and mineral transporters among cell types

Current models of normal homeostatic fluid transport propose that substantial ion and ion‐coupled nutrient absorption by enterocytes along the villus drives water absorption, negating modest fluid secretion occurring in the crypt region (Thiagarajah et al., 2018). In secretory diarrhea, Cl^–^ secretion via CFTR in crypt cells is dramatically enhanced by cAMP, markedly increasing crypt fluid secretion that results in net fluid loss even though fluid absorption in the villus region remains normal (Petri et al., 2008). Our findings that the mRNA of important ion transporters are distributed appropriately in specific cell types are supported by previous immunocytochemical localization of NBCe1 and DRA in the villus region lined by enterocytes, with CFTR and NKCC1 mainly in intestinal crypts containing PAN and ISC (Foulke‐Abel et al., 2016; Jakab et al., 2011). NKCC1 is also highly expressed in crypt‐like, undifferentiated but not in villus‐like differentiated human enteroids (Foulke‐Abel et al., 2016). It is not clear how co‐expression of ACE2 with NBCe1 and DRA in villus‐dwelling cells, or with CFTR and NKCC1 in crypt‐dwelling cells affect ACE2 regulation by chloride and sodium (Post et al., 2020; Rushworth et al., 2008). Its basolateral location enables NKCC1 to transport sodium, potassium, and chloride from the blood into the cell, allowing electrogenic chloride secretion by CFTR across the apical membrane to the lumen followed by cations and then fluids (Thiagarajah et al., 2018). Thus, the membrane location and expression of NKCC1 in crypt‐dwelling cells reflect its prominent role in intestinal fluid secretion.

Protein expression of DMT1, FTL, IREG1, and HEPH has been localized primarily in villus‐residing cells (Fuqua et al., 2014; Yeh et al., 2011). As expected, significant mRNA levels of genes involved in iron transport were expressed in villus‐residing ENT already known to regulate nutritional iron needs. It is not clear why other cell types express such high mRNA levels of these genes, because the sugar transporters GLUT5, GLUT2, and SGLT1 are much greater in ENT and GOB compared to ISC and PAN (Kishida et al., 2017). GOB cells have been observed to transport iron in human intestine (Refsum & Schreiner, 1980) while bactericidal PAN cells may transport iron from the crypt lumen to reduce luminal bacterial load as bacteria and viruses (including SARS‐CoV‐2) depend on iron to thrive (Kortman et al., 2014; Liu et al., 2020). Moreover, iron regulates levels of antimicrobial peptides that PAN cells secrete. ISC may express relatively significant levels of iron transporters, not only because iron is required for intestinal epithelial proliferation and homeostasis (Chen et al., 2015) but also because crypt cells regulate systemic iron status by sensing levels of iron‐occupied transferrin in the blood (Pietrangelo, 2002).

TRPV6 is an epithelial calcium channel mediating dietary calcium entry into intestinal cells then PMCA1 is an ATPase that extrudes calcium from the cytosol to the blood. In rodents whose calcium status is normal, we have previously shown that entry factors for intestinal calcium absorption are repressed (Douard et al., 2014). Thus, the relative expression of TRPV6 is low in ENT as these were obtained from calcium‐replete adult mice with minimal calcium requirements. Since calcium ions increase cell infectivity to corona viruses (Straus et al., 2020), downregulation of TRPV6 levels in the gut should be helpful. Our findings of high levels of mRNA expression of PMCA1 in most cell types confirm previous work indicating that PMCA1 has higher expression and activity in cells along the villus (Centeno et al., 2004).

### 4.4 Perspective and limitations

Unlike electrolyte transporters whose marked heterogeneous expression in specific cell types and membranes is strategically localized to promote net intestinal fluid absorption, entry factors of SARS‐CoV‐2 are expressed significantly in virtually all cell types. This information, coupled with literature reports of their expression in all intestinal regions, suggests that this virus has the potential to enter any mucosal cell from the duodenum to the distal colon, explaining in part its virulence. However, significantly higher numbers of enterocytes in vivo compared to the other lineages studied underlie the relative importance of this cell type for viral entry. While SARS‐CoV‐2 spike protein is a poor ligand of mouse ACE2, the remaining primary structure of ACE2 protein likely regulating function and cell type distribution is highly conserved and in rodents is phylogenetically closer to that of primates when compared to those of other mammalian and bird families (Johansen et al., 2020). This suggests that cell type distribution of ACE2 may probably be similar for mice and humans.

While both species possess the same transporters and genes associated with SARS‐CoV‐2 and secretory diarrhea, whose intestinal cell type distributions are now known, there are limitations to the interpretation of these findings because of inherent complexities in recreating human diarrheal symptoms in mice.

In this study, we guided the strict differentiation of cultured intestinal epithelial enteroids so they can be enriched with their assigned cell types (Kishida et al., 2017; Yin et al., 2014) and allowed us to compare, in different cell types, the expression of transporters involved in ion transport to that of SARS‐CoV‐2 entry. While the small number of cells in these enteroids limit readouts to mRNA expression, our new findings allow for a better characterization of the capabilities of PAN, GOB, and ISC found in relatively small proportions in the gut mucosa. Although each enteroid type representing a specific cell cannot be created as to be comprised purely of that cell type, it does contain a much greater proportion of that single cell type compared with that in vivo in the small intestine. Moreover, there are a number of proteases proposed to participate in intestinal cellular entry of SARS‐CoV‐2, but because of limited amount of material from organoids, we had to prioritize our list of entry co‐factors to those most widely, if not universally cited, like ACE2, TMPRSS2 and 4, furin, and ADAM17. Although enteroids were induced to differentiate almost exclusively to a specific epithelial lineage, studies are typically not conducted in enteroids forcibly differentiated to, for example, goblet and Paneth cell lineages, so that it is not at all clear that these models necessarily reflect the transporter properties of these lineages when present at their natural abundances, where the gene expression of a given lineage might well be influenced by paracrine interactions with neighboring cells (readers are referred to comprehensive reports on numerous transporters from the (Foulke‐Abel et al., 2016) and (Turner, 2020) laboratories regarding comparative expression of various transporters in intact intestinal tissues and enteroids.

## DISCLOSURES

No conflict of interest.

## AUTHOR CONTRIBUTIONS

S.C.P. conducted the experiments, analyzed the data, helped with the experimental design, and prepared the manuscript; P.S., K.K., A. A., I.N., R.S., J.F., and M.A. conducted the experiments and analyzed the data; S.Y. and N.G. helped to design the experiments, analyzed the data, and revised the manuscript; R.P.F. conceptualized and designed the project, and finalized the manuscript.

## References

[phy215061-bib-0001] Barbosa da Luz, B. , de Oliveira, N. M. T. , França Dos Santos, I. W. , Paza, L. Z. , Braga, L. , Platner, F. D. S. , Werner, M. F. P. , Fernandes, E. S. , & Maria‐Ferreira, D. (2020). An overview of the gut side of the SARS‐CoV‐2 infection. Intestinal Research. 10.5217/ir.2020.00087 PMC8566822 33142370

[phy215061-bib-0002] Burgueno, J. F. , Reich, A. , Hazime, H. , Quintero, M. A. , Fernandez, I. , Fritsch, J. , Santander, A. M. , Brito, N. , Damas, O. M. , Deshpande, A. , Kerman, D. H. , Zhang, L. , Gao, Z. , Ban, Y. , Wang, L. , Pignac‐Kobinger, J. , & Abreu, M. T. (2020). Expression of SARS‐CoV‐2 entry molecules ACE2 and TMPRSS2 in the gut of patients with IBD. Inflammatory Bowel Diseases, 26, 797–808. 10.1093/ibd/izaa085 32333601 PMC7188157

[phy215061-bib-0003] Camargo, S. M. R. , Vuille‐Dit‐Bille, R. N. , Meier, C. F. , & Verrey, F. (2020). ACE2 and gut amino acid transport. Clinical Science (Lond), 134, 2823–2833. 10.1042/CS20200477 33140827

[phy215061-bib-0004] Centeno, V. A. , Diaz de Barboza, G. E. , Marchionatti, A. M. , Alisio, A. E. , Dallorso, M. E. , Nasif, R. , & Tolosa de Talamoni, N. G. (2004). Dietary calcium deficiency increases Ca2+ uptake and Ca2+ extrusion mechanisms in chick enterocytes. Comparative Biochemistry and Physiology Part A Molecular Integrative Physiology, 139, 133–141. 10.1016/j.cbpb.2004.08.002 15528161

[phy215061-bib-0005] Chen, A. C. , Donovan, A. , Ned‐Sykes, R. , & Andrews, N. C. (2015). Noncanonical role of transferrin receptor 1 is essential for intestinal homeostasis. Proceedings of the National Academy of Sciences of the United States of America, 112, 11714–11719. 10.1073/pnas.1511701112 26324903 PMC4577163

[phy215061-bib-0006] Cheng, H. , & Origin, L. C. P. (1974). Origin differentiation and renewal of the four main epithelial cell types in the mouse small intestine. V. Unitarian theory of the origin of the four epithelial cell types. The American Journal of Anatomy, 141, 537–561. 10.1002/aja.1001410407 4440635

[phy215061-bib-0007] Damas, J. , Hughes, G. M. , Keough, K. C. , Painter, C. A. , Persky, N. S. , Corbo, M. , Hiller, M. , Koepfli, K. P. , Pfenning, A. R. , Zhao, H. , Genereux, D. P. , Swofford, R. , Pollard, K. S. , Ryder, O. A. , Nweeia, M. T. , Lindblad‐Toh, K. , Teeling, E. C. , Karlsson, E. K. , & Lewin, H. A. (2020). Broad host range of SARS‐CoV‐2 predicted by comparative and structural analysis of ACE2 in vertebrates. Proceedings of the National Academy of Sciences of the United States of America, 117, 22311–22322. 32826334 10.1073/pnas.2010146117 PMC7486773

[phy215061-bib-0008] Deng, N. , Puetter, A. , Zhang, K. , Johnson, K. , Zhao, Z. , Taylor, C. , Flemington, E. K. , & Zhu, D. (2011). Isoform‐level microRNA‐155 target prediction using RNA‐seq. Nucleic Acids Research, 39, e61. 10.1093/nar/gkr042 21317189 PMC3089486

[phy215061-bib-0009] Douard, V. , Patel, C. , Lee, J. , Tharabenjasin, P. , Williams, E. , Fritton, J. C. , Sabbagh, Y. , & Ferraris, R. P. (2014). Chronic high fructose intake reduces serum 1,25 (OH)2D3 levels in calcium‐sufficient rodents. PLoS One, 9, e93611. 10.1371/journal.pone.0093611 24718641 PMC3981704

[phy215061-bib-0010] Foulke‐Abel, J. , In, J. , Yin, J. , Zachos, N. C. , Kovbasnjuk, O. , Estes, M. K. , de Jonge, H. , & Donowitz, M. (2016). Human enteroids as a model of upper small intestinal ion transport physiology and pathophysiology. Gastroenterology, 150, 638–649.e8. 10.1053/j.gastro.2015.11.047 26677983 PMC4766025

[phy215061-bib-0011] Fuqua, B. K. , Lu, Y. , Darshan, D. , Frazer, D. M. , Wilkins, S. J. , Wolkow, N. , Bell, A. G. , Hsu, J. , Yu, C. C. , Chen, H. , Dunaief, J. L. , Anderson, G. J. , & Vulpe, C. D. (2014). The multicopper ferroxidase hephaestin enhances intestinal iron absorption in mice. PLoS One, 9, e98792. 10.1371/journal.pone.0098792 24896847 PMC4045767

[phy215061-bib-0012] Gerbe, F. , van Es, J. H. , Makrini, L. , Brulin, B. , Mellitzer, G. , Robine, S. , Romagnolo, B. , Shroyer, N. F. , Bourgaux, J. F. , Pignodel, C. , Clevers, H. , & Jay, P. (2011). Distinct ATOH1 and Neurog3 requirements define tuft cells as a new secretory cell type in the intestinal epithelium. Journal of Cell Biology, 192, 767–780. 10.1083/jcb.201010127 PMC3051826 21383077

[phy215061-bib-0013] Gheblawi, M. , Wang, K. , Viveiros, A. , Nguyen, Q. , Zhong, J. C. , Turner, A. J. , Raizada, M. K. , Grant, M. B. , & Oudit, G. Y. (2020). Angiotensin‐converting enzyme 2: SARS‐CoV‐2 receptor and regulator of the Renin‐Angiotensin system: Celebrating the 20th anniversary of the discovery of ACE2. Circulation Research, 126, 1456–1474. 32264791 10.1161/CIRCRESAHA.120.317015 PMC7188049

[phy215061-bib-0014] Guney, C. , & Akar, F. (2021). Epithelial and endothelial expressions of ACE2: SARS‐CoV‐2 entry routes. Journal of Pharmacy and Pharmaceutical Sciences, 24, 84–93. 33626315 10.18433/jpps31455

[phy215061-bib-0015] Hamming, I. , Timens, W. , Bulthuis, M. L. , Lely, A. T. , Navis, G. , & van Goor, H. (2004). Tissue distribution of ACE2 protein, the functional receptor for SARS coronavirus. A first step in understanding SARS pathogenesis. The Journal of Pathology, 203, 631–637. 10.1002/path.1570 15141377 PMC7167720

[phy215061-bib-0016] Hoffmann, M. , Kleine‐Weber, H. , Schroeder, S. , Kruger, N. , Herrler, T. , Erichsen, S. , Schiergens, T. S. , Herrler, G. , Wu, N. H. , Nitsche, A. , Muller, M. A. , Drosten, C. , & Pohlmann, S. (2020). SARS‐CoV‐2 cell entry depends on ACE2 and TMPRSS2 and is blocked by a clinically proven protease inhibitor. Cell, 181, 271–280.e8. 10.1016/j.cell.2020.02.052 32142651 PMC7102627

[phy215061-bib-0017] Jakab, R. L. , Collaco, A. M. , & Ameen, N. A. (2011). Physiological relevance of cell‐specific distribution patterns of CFTR, NKCC1, NBCe1, and NHE3 along the crypt‐villus axis in the intestine. American Journal of Physiology. Gastrointestinal and Liver Physiology, 300, G82–G98. 10.1152/ajpgi.00245.2010 21030607 PMC3025502

[phy215061-bib-0018] Jando, J. , Camargo, S. M. R. , Herzog, B. , & Verrey, F. (2017). Expression and regulation of the neutral amino acid transporter B0AT1 in rat small intestine. PLoS One, 12, e0184845. 10.1371/journal.pone.0184845 28915252 PMC5600382

[phy215061-bib-0019] Jia, H. , Yue, X. , & Lazartigues, E. (2020). ACE2 mouse models: a toolbox for cardiovascular and pulmonary research. Nature Communications, 11, 5165. 10.1038/s41467-020-18880-0 PMC7560817 33057007

[phy215061-bib-0020] Johansen, M. D. , Irving, A. , Montagutelli, X. , Tate, M. D. , Rudloff, I. , Nold, M. F. , Hansbro, N. G. , Kim, R. Y. , Donovan, C. , Liu, G. , Faiz, A. , Short, K. R. , Lyons, J. G. , McCaughan, G. W. , Gorrell, M. D. , Cole, A. , Moreno, C. , Couteur, D. , Hesselson, D. , … Hansbro, P. M. (2020). Animal and translational models of SARS‐CoV‐2 infection and COVID‐19. Mucosal Immunology, 13, 877–891. 10.1038/s41385-020-00340-z 32820248 PMC7439637

[phy215061-bib-0021] Kishida, K. , Pearce, S. C. , Yu, S. , Gao, N. , & Ferraris, R. P. (2017). Nutrient sensing by absorptive and secretory progenies of small intestinal stem cells. American Journal of Physiology. Gastrointestinal and Liver Physiology, 312, G592–G605. 10.1152/ajpgi.00416.2016 28336548 PMC5495913

[phy215061-bib-0022] Koester, S. T. , Li, N. , Lachance, D. M. , Morella, N. M. , & Dey, N. (2021). Variability in digestive and respiratory tract Ace2 expression is associated with the microbiome. PLoS One, 16, e0248730. 10.1371/journal.pone.0248730 33725024 PMC7963026

[phy215061-bib-0023] Kortman, G. A. , Raffatellu, M. , Swinkels, D. W. , & Tjalsma, H. (2014). Nutritional iron turned inside out: intestinal stress from a gut microbial perspective. FEMS Microbiology Reviews, 38, 1202–1234. 25205464 10.1111/1574-6976.12086

[phy215061-bib-0024] Kowalczuk, S. , Broer, A. , Tietze, N. , Vanslambrouck, J. M. , Rasko, J. E. , & Broer, S. (2008). A protein complex in the brush‐border membrane explains a Hartnup disorder allele. The FASEB Journal, 22, 2880–2887. 10.1096/fj.08-107300 18424768

[phy215061-bib-0025] Lam, S. D. , Bordin, N. , Waman, V. P. , Scholes, H. M. , Ashford, P. , Sen, N. , van Dorp, L. , Rauer, C. , Dawson, N. L. , Pang, C. S. M. , Abbasian, M. , Sillitoe, I. , Edwards, S. J. L. , Fraternali, F. , Lees, J. G. , Santini, J. M. , & Orengo, C. A. (2020). SARS‐CoV‐2 spike protein predicted to form complexes with host receptor protein orthologues from a broad range of mammals. Scientific Reports, 10, 16471. 10.1038/s41598-020-71936-5 33020502 PMC7536205

[phy215061-bib-0026] Lamers, M. M. , Beumer, J. , van der Vaart, J. , Knoops, K. , Puschhof, J. , Breugem, T. I. , Ravelli, R. B. G. , Paul van Schayck, J. , Mykytyn, A. Z. , Duimel, H. Q. , van Donselaar, E. , Riesebosch, S. , Kuijpers, H. J. H. , Schipper, D. , van de Wetering, W. J. , de Graaf, M. , Koopmans, M. , Cuppen, E. , Peters, P. J. , … Clevers, H. (2020). SARS‐CoV‐2 productively infects human gut enterocytes. Science, 369, 50–54. 10.1126/science.abc1669 32358202 PMC7199907

[phy215061-bib-0027] Li, R. , Qiao, S. , & Zhang, G. (2020). Analysis of angiotensin‐converting enzyme 2 (ACE2) from different species sheds some light on cross‐species receptor usage of a novel coronavirus 2019‐nCoV. Journal of Infection, 80, 469–496. 10.1016/j.jinf.2020.02.013 PMC7127620 32092392

[phy215061-bib-0028] Lippi, G. , South, A. M. , & Henry, B. M. (2020). Electrolyte imbalances in patients with severe coronavirus disease 2019 (COVID‐19). Annals of Clinical Biochemistry, 57, 262–265. 10.1177/0004563220922255 32266828 PMC8173320

[phy215061-bib-0029] Liu, W. , Zhang, S. , Nekhai, S. , & Liu, S. (2020). Depriving iron supply to the virus represents a promising adjuvant therapeutic against viral survival. Current Clinical Microbiology Reports, 7, 1–7. 32318324 10.1007/s40588-020-00140-w PMC7169647

[phy215061-bib-0030] Montgomery, R. K. , & Breault, D. T. (2008). Small intestinal stem cell markers. Journal of Anatomy, 213, 52–58. 10.1111/j.1469-7580.2008.00925.x 18638070 PMC2475558

[phy215061-bib-0031] Pearce, S. C. , Al‐Jawadi, A. , Kishida, K. , Yu, S. , Hu, M. , Fritzky, L. F. , Edelblum, K. L. , Gao, N. , & Ferraris, R. P. (2018). Marked differences in tight junction composition and macromolecular permeability among different intestinal cell types. BMC Biology, 16, 19. 10.1186/s12915-018-0481-z 29391007 PMC5793346

[phy215061-bib-0032] Peng, J. B. , Suzuki, Y. , Gyimesi, G. , & Hediger, M. A. (2018). TRPV5 and TRPV6 Calcium‐Selective Channels. In: J. A. Kozak , & J. W. Putney Jr (Eds.), Calcium entry channels in non‐excitable cells (pp. 241–274). 30299660

[phy215061-bib-0033] Petri, W. A. Jr , Miller, M. , Binder, H. J. , Levine, M. M. , Dillingham, R. , & Guerrant, R. L. (2008). Enteric infections, diarrhea, and their impact on function and development. Journal of Clinical Investigation, 118, 1277–1290. 10.1172/JCI34005 PMC2276781 18382740

[phy215061-bib-0034] Pietrangelo, A. (2002). Physiology of iron transport and the hemochromatosis gene. American Journal of Physiology. Gastrointestinal and Liver Physiology, 282, G403–G414. 10.1152/ajpgi.00404.2001 11841990

[phy215061-bib-0035] Pietrangelo, A. (2010). Hereditary hemochromatosis: pathogenesis, diagnosis, and treatment. Gastroenterology 139, 393–408(408), e391–e392. 10.1053/j.gastro.2010.06.013 20542038

[phy215061-bib-0036] Post, A. , Dullaart, R. P. F. , & Bakker, S. J. L. (2020). Sodium status and kidney involvement during COVID‐19 infection. Virus Research, 286, 198034. 10.1016/j.virusres.2020.198034 32445872 PMC7240271

[phy215061-bib-0037] Refsum, S. B. , & Schreiner, B. (1980). Iron excretion from the goblet cells of the small intestine in man. An additional regulatory mechanism in iron homeostasis? Scandinavian Journal of Gastroenterology, 15, 1013–1020. 10.3109/00365528009181806 6940236

[phy215061-bib-0038] Rushworth, C. A. , Guy, J. L. , & Turner, A. J. (2008). Residues affecting the chloride regulation and substrate selectivity of the angiotensin‐converting enzymes (ACE and ACE2) identified by site‐directed mutagenesis. FEBS Journal, 275, 6033–6042. 10.1111/j.1742-4658.2008.06733.x PMC7163990 19021774

[phy215061-bib-0039] Straus, M. R. , Tang, T. , Lai, A. L. , Flegel, A. , Bidon, M. , Freed, J. H. , Daniel, S. , & Whittaker, G. R. (2020). Ca2+ ions promote fusion of Middle East Respiratory Syndrome coronavirus with host cells and increase infectivity. Journal of Virology, 94, e00426–20. 10.1128/JVI.00426-20 32295925 PMC7307142

[phy215061-bib-0040] Thiagarajah, J. R. , & Verkman, A. S. (2018). Water transport in the gastrointestinal tract. In: H. M. Said (Ed.), Physiology of the gastrointestinal tract (pp. 1249–1272). Academic press.

[phy215061-bib-0041] Turner, J. R. (2020). Atlas of intestinal transport. Laboratory of Mucosal Barrier Pathobiology, Harvard Medical School. https://jrturnerlab.com/database‐viewer/atlas‐of‐intestinal‐transport/

[phy215061-bib-0042] Umar, S. (2010). Intestinal stem cells. Current Gastroenterology Reports, 12, 340–348. 10.1007/s11894-010-0130-3 20683682 PMC2965634

[phy215061-bib-0043] Vargas‐Vargas, M. , & Cortes‐Rojo, C. (2020). Ferritin levels and COVID‐19. Revista Panamericana De Salud Publica, 44, e72. 10.26633/RPSP.2020.72 32547616 PMC7286435

[phy215061-bib-0044] Vuille‐dit‐Bille, R. N. , Camargo, S. M. , Emmenegger, L. , Sasse, T. , Kummer, E. , Jando, J. , Hamie, Q. M. , Meier, C. F. , Hunziker, S. , Forras‐Kaufmann, Z. , Kuyumcu, S. , Fox, M. , Schwizer, W. , Fried, M. , Lindenmeyer, M. , Gotze, O. , & Verrey, F. (2015). Human intestine luminal ACE2 and amino acid transporter expression increased by ACE‐inhibitors. Amino Acids, 47, 693–705. 10.1007/s00726-014-1889-6 25534429

[phy215061-bib-0045] Wagener, F. , Pickkers, P. , Peterson, S. J. , Immenschuh, S. , & Abraham, N. G. (2020). Targeting the Heme‐Heme oxygenase system to prevent severe complications following COVID‐19 infections. Antioxidants, 9, 540 10.3390/antiox9060540 PMC7346191 32575554

[phy215061-bib-0046] Wu, Y. , Hou, B. , Liu, J. , Chen, Y. , & Zhong, P. (2020). Risk factors associated with long‐term hospitalization in patients with COVID‐19: A single‐centered, retrospective study. Frontiers in Medicine (Lausanne), 7, 315. 10.3389/fmed.2020.00315 PMC7296106 32582749

[phy215061-bib-0047] Xiao, F. , Tang, M. , Zheng, X. , Liu, Y. , Li, X. , & Shan, H. (2020). Evidence for Gastrointestinal Infection of SARS‐CoV‐2. Gastroenterology, 158, 1831–1833.e3. 10.1053/j.gastro.2020.02.055 32142773 PMC7130181

[phy215061-bib-0048] Yeh, K. Y. , Yeh, M. , Polk, P. , & Glass, J. (2011). Hypoxia‐inducible factor‐2alpha and iron absorptive gene expression in Belgrade rat intestine. American Journal of Physiology. Gastrointestinal and Liver Physiology, 301, G82–G90. 21436314 10.1152/ajpgi.00538.2010 PMC3129931

[phy215061-bib-0049] Yin, X. , Farin, H. F. , van Es, J. H. , Clevers, H. , Langer, R. , & Karp, J. M. (2014). Niche‐independent high‐purity cultures of Lgr5(+) intestinal stem cells and their progeny. Nature Methods, 11, 106–112. 10.1038/nmeth.2737 24292484 PMC3951815

[phy215061-bib-0050] Zang, R. , Gomez Castro, M. F. , McCune, B. T. , Zeng, Q. , Rothlauf, P. W. , Sonnek, N. M. , Liu, Z. , Brulois, K. F. , Wang, X. , Greenberg, H. B. , Diamond, M. S. , Ciorba, M. A. , Whelan, S. P. J. , & Ding, S. (2020). TMPRSS2 and TMPRSS4 promote SARS‐CoV‐2 infection of human small intestinal enterocytes. Science Immunology, 5, eabc3582. 32404436 10.1126/sciimmunol.abc3582 PMC7285829

[phy215061-bib-0051] Zhang, H. , Li, H. B. , Lyu, J. R. , Lei, X. M. , Li, W. , Wu, G. , Lyu, J. , & Dai, Z. M. (2020). Specific ACE2 expression in small intestinal enterocytes may cause gastrointestinal symptoms and injury after 2019‐nCoV infection. International Journal of Infectious Diseases, 96, 19–24. 10.1016/j.ijid.2020.04.027 32311451 PMC7165079

[phy215061-bib-0052] Zhao, K. , Huang, J. , Dai, D. , Feng, Y. , Liu, L. , & Nie, S. (2019). Serum iron level as a potential predictor of coronavirus disease 2019 severity and mortality: A retrospective study. Open Forum Infectious Diseases, 7, ofaa250. 10.1093/ofid/ofaa250 PMC7337740 32661499

